# Supplementary material for: Identification and functional analysis of non-coding regulatory small RNA FenSr3 in Bacillus amyloliquefaciens LPB-18
Source: PeerJ. 2023 May 15;11:e15236. doi: 10.7717/peerj.15236 (PMC10194069; doi:10.7717/peerj.15236)
Supplement: Supplemental Information 4 [file peerj-11-15236-s004.zip › KO/CK-vs-T1_map/map00950.html]

KEGG PATHWAY: Isoquinoline alkaloid biosynthesis - Reference pathway


|  |  |
| --- | --- |
| **Isoquinoline alkaloid biosynthesis - Reference pathway** |  |

[
Pathway menu
| Organism menu
| Pathway entry
| Show description
| User data mapping
]

Isoquinoline alkaloids are tyrosine-derived plant alkaloids with an isoquinoline skeleton. Among them benzylisoquinoline alkaloids form an important group with potent pharmacological activity, including analgesic compounds of morphine and codeine, and anti-infective agents of berberine, palmatine, and magnoflorine. Biosynthesis of isoquinoline alkaloids proceeds via decarboxylation of tyrosine or DOPA to yield dopamine, which together with 4-hydroxyphenylacetaldehyde, an aldehyde derived from tyrosine, is converted to reticuline, an important precursor of various benzylisoquinoline alkaloids.

|  |  |  |
| --- | --- | --- |
| Reference pathway | 184% 150% 122% 100% 82% 67% 55% | 图片下载 |
